# Molecular Characterization of Non‐Specific Esophagitis Reveals a Distinct Non‐Eosinophilic Phenotype With Fibrotic Traits

**DOI:** 10.1111/all.70364

**Published:** 2026-05-01

**Authors:** Marc Pfefferle, Darko Stojkov, Dagmar Simon, Emilie Gueguen, Marcin Wawrzyniak, Margaret H. Collins, Mirna Chehade, Evan S. Dellon, Glenn T. Furuta, Ikuo Hirano, Nirmala Gonsalves, Fouad J. Moawad, Luc Biedermann, Alain M. Schoepfer, Alex Straumann, Hans‐Uwe Simon, Thomas Greuter

**Affiliations:** ^1^ Department of Gastroenterology and Hepatology University Hospital Zurich Zurich Switzerland; ^2^ Division of Gastroenterology and Hepatology University Hospital Lausanne—Centre Hospitalier Universitaire Vaudois (CHUV) Lausanne Switzerland; ^3^ Department of Internal Medicine University Hospital Lausanne—Centre Hospitalier Universitaire Vaudois (CHUV) Lausanne Switzerland; ^4^ Department of Internal Medicine Limmattalspital Schlieren Switzerland; ^5^ Institute of Pharmacology University of Bern Bern Switzerland; ^6^ Department of Dermatology, Inselspital, Bern University Hospital University of Bern Bern Switzerland; ^7^ Division of Gastroenterology and Hepatology EOC Lugano Lugano Switzerland; ^8^ Faculty of Biomedical Sciences Università Della Svizzera Italiana Lugano Switzerland; ^9^ Division of Pathology, Cincinnati Children's Hospital Medical Center, Department of Pathology University of Cincinnati College of Medicine Cincinnati Ohio USA; ^10^ Mount Sinai Center for Eosinophilic Disorders Icahn School of Medicine at Mount Sinai New York New York USA; ^11^ Center for Esophageal Diseases and Swallowing, Division of Gastroenterology and Hepatology University of North Carolina School of Medicine Chapel Hill North Carolina USA; ^12^ Department of Pediatrics, Gastrointestinal Eosinophilic Diseases Program University of Colorado School of Medicine, Digestive Health Institute, Children's Hospital Colorado Aurora Colorado USA; ^13^ Division of Gastroenterology and Hepatology Northwestern University Chicago Illinois USA; ^14^ Division of Gastroenterology Scripps Clinic La Jolla California USA; ^15^ Institute of Biochemistry Brandenburg Medical School Neuruppin Germany

## Abstract

**Background:**

Non‐specific esophagitis has been recently described in patients with dysphagia not fulfilling criteria for eosinophilic esophagitis (EoE). Whether it is a distinct entity and how dysphagia can develop in the absence of eosinophilic infiltration remains unknown.

**Objective:**

In this study, we comprehensively characterized non‐specific esophagitis aiming to elucidate eosinophil‐independent fibrosis mechanisms.

**Methods:**

We cross‐sectionally and longitudinally analyzed treatment‐naïve patients presenting with non‐specific esophagitis defined by esophageal dysfunction and low‐grade lymphocytic infiltration (< 30 lymphocytes/hpf) in the absence of tissue eosinophilia and reflux disease (GERD). We compared clinical, endoscopic, (immuno)‐histological disease activity to EoE, lymphocytic esophagitis, and GERD. RNA sequencing was performed to investigate disease mechanisms.

**Results:**

We identified 19 patients (6 males, median age 48 years), with a follow‐up of 20 months (IQR 10–41). Clinical disease burden was considerable, contrasting mild endoscopic activity (EREFS 0, IQR 0–2). Immunostaining of esophageal biopsies did not reveal increased numbers of eosinophils or mast cells as contributors to the disease. Lymphocytic infiltration (15/hpf, IQR 9–24) was not increased compared to healthy controls (9/hpf, IQR 9–10) or GERD patients (13/hpf, IQR 9–23); 14 (73.6%) patients were treated with topical steroids (symptomatic response in 85.7%) and 6 patients (31.6%) underwent endoscopic dilation. Progression to EoE was observed in 1 patient, while esophageal eosinophilia (< 15 eos/hpf) developed in 3 patients. RNA sequencing (*n* = 10) revealed a distinct transcriptomic profile, with highly enriched fibrosis‐related pathways. Immunostaining for E‐Cadherin/Vimentin as a marker for epithelial‐mesenchymal transition (EMT, *n* = 10) confirmed increased EMT compared to controls.

**Conclusion:**

Non‐specific esophagitis appears to be a distinct non‐eosinophilic phenotype with fibrotic traits. Our data suggest eosinophil‐independent development of fibrosis.

## Graphical abstract

This study comprehensively characterizes non‐specific esophagitis and elucidates eosinophil‐independent fibrosis mechanisms. NSE appears to be a distinct fibrotic phenotype of the esophagus, without any considerable overlap with GERD and other chronic inflammatory disorders such as EoE or lymphocytic esophagitis. Our data suggest development of fibrotic traits independently of inflammatory changes, in particular tissue eosinophilia. EoE, eosinophilic esophagitis; FBLN5, fibulin‐5; GERD, gastroesophageal reflux disease; HPF, high‐power field; IF, immunofluorescence; LyE, lymphocytic esophagitis; NSE, non‐specific esophagitis; PCA, principal component analysis.

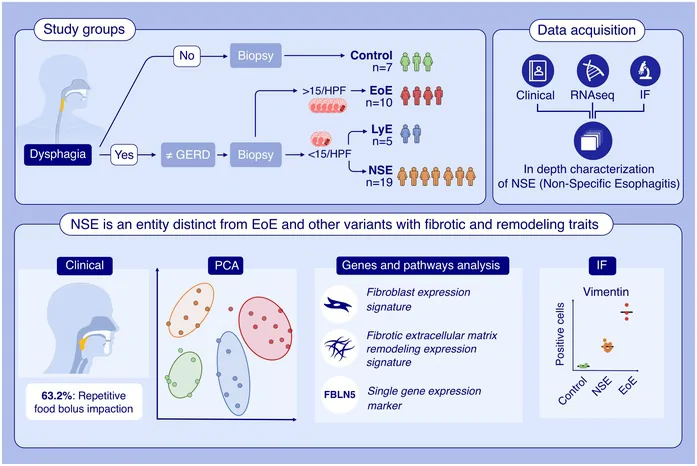

## 1 Introduction

Non‐specific esophagitis has been recently described in patients presenting with symptoms suggestive of eosinophilic esophagitis (EoE), but absence of eosinophilic infiltration. It stands out for its low‐grade inflammation, defined by an infiltration with lymphocytes below the threshold for lymphocytic esophagitis, and by the absence of typical EoE features, including eosinophil infiltration [1, 2]. However, the clinical presentation, natural history and molecular underpinnings of non‐specific esophagitis remain poorly understood. Standard histological assessment provides little insight into the pathophysiology of this condition, and published data to date suggest a distinct entity that lacks typical TSLP or eotaxin‐3 expression [2]. This raises the possibility that symptoms in these patients arise from alternative pathogenic mechanisms, such as tissue remodeling and fibrosis, independently of eosinophilic inflammation. Whether fibrosis can occur in the absence of eosinophil‐driven inflammation, however, remains unknown.

Fibrosis is a considerable problem in EoE, where long‐standing eosinophilic inflammation almost uniformly results in the development of fibrostenosis [3]. Current data suggest a co‐presence of inflammation and fibrosis rather than development of fibrosis independently of inflammation [4, 5]. Late‐stage inflammatory cells such as eosinophils and mast cells have been linked to the activation of fibroblasts [6] but fibrosis can also occur relatively early in the EoE disease course through other mechanisms such as epithelial‐mesenchymal transition (EMT) [7]. However, the exact mechanisms of fibrosis in EoE remain unknown. In particular, it remains elusive whether eosinophilic infiltration is a requirement for fibrosis development or if fibrosis can develop independently. Thus, characterization of fibrotic changes in non‐specific esophagitis would not only contribute to the understanding of this potentially novel disease entity but would unravel fibrosis mechanisms in the esophagus in general. This is of importance given the lack of efficacious anti‐fibrotic treatment.

To address these knowledge gaps, we sought to characterize the clinical course and molecular features of non‐specific esophagitis, with a particular focus on tissue remodeling and fibrosis. Here, we present a systematic longitudinal analysis of patients with non‐specific esophagitis, with detailed pathway enrichment analyses of RNA sequencing data and in‐depth immunostaining analyses studying EMT and epithelial barrier function. Thereby, we aimed at answering the question of whether it is a distinct phenotype and if fibrosis can occur independently of tissue eosinophilia in this condition.

## 2 Methods

### 2.1 Study Design

This work is an analysis of an observational longitudinal multicenter study involving adult patients with non‐specific esophagitis, included in the EoE variant database [2]. This database comprises patients from 6 EoE referral centers in Switzerland (*n* = 1) and the United States (*n* = 5). For the purpose of the current study, we analyzed clinical, endoscopic, histological, immunohistochemical and molecular data of patients with non‐specific esophagitis. Esophagus healthy individuals, EoE patients, patients with lymphocytic esophagitis and gastro‐esophageal reflux disease (GERD) patients also included in the database served as controls. The study was approved by the local ethics committee of each of the participating centers (EKNZ 2015‐388/CER‐VD 148‐15, COMIRB 07‐0888, UNC IRB 14‐1442, IRB‐16‐6910, STU00094108, ISMMS HS‐10‐00070), with available written informed consent from all patients.

### 2.2 Patients and Data Collection

The EoE variant database includes patients presenting with symptoms compatible with EoE, but absence of esophageal eosinophilia defined by a cutoff of 15 eosinophils per hpf (in the absence of anti‐eosinophil treatment or dietary restrictions and in the absence of GERD) for which baseline esophageal biopsies were obtained. Patients on topical corticosteroids (inhaled corticosteroids and nasal corticosteroids) at baseline were excluded as were patients with other esophageal, systemic, or fibrotic diseases. For the purpose of this study, only patients classified as having non‐specific esophagitis were analyzed. Non‐specific esophagitis has been previously defined [2] and is characterized by a histological infiltration of lymphocytes not fulfilling the numerical and distributional criteria of lymphocytic esophagitis, in the absence of tissue eosinophilia, corresponding to a low‐grade inflammation. Esophagus‐healthy controls were patients that underwent upper endoscopy for non‐esophageal symptoms without detection of changes within the esophagus, while GERD patients had been previously diagnosed with reflux disease without the presence of another esophageal disease. Control patients (esophagus‐healthy individuals, EoE patients, and lymphocytic esophagitis patients) were recruited from the same centers over the same time frame. The structured data collection was performed by means of a standardized spreadsheet. All data were anonymized. For details see Supporting Information.

### 2.3 Scores

The following disease scores were used:

- EoE Endoscopic Reference Score to assess the following endoscopic alterations: (1) mucosal edema (absent = 0; present = 1), (2) rings (absent = 0; mild = 1; moderate = 2; severe = 3), (3) white exudates (absent = 0; mild = 1; severe = 2), (4) furrows (absent = 0; mild = 1; 2 = severe) and (5) strictures (absent = 0; present = 1) [8].
- EoE‐HSS: EoE‐HSS grading and staging scores were calculated as follows: scores in each individual component were added and divided through the maximum possible score (e.g., 21 if lamina propria is not assessed, 24 if all components including lamina propria are evaluated) and multiplied by 100 to give a score range from 0 to 100 [9].
- Disease severity judged by patients and treating physician based on a numerical rating scale for symptom assessment from 0 to 10, with 0 indicating no symptoms and 10 indicating the most severe disease.

### 2.4 Histological Re‐Examination and Immunostaining

Histological re‐examination of all available biopsies was performed as previously described [2]. Briefly, all biopsies were analyzed using the EoE‐HSS [9]. In addition, peak lymphocyte counts were reported. For a more detailed analysis, immunostaining was performed using formalin‐fixed, paraffin‐embedded esophageal biopsies as previously described [2]. Briefly, EPX‐positive peak counts per hpf (eosinophils), CD3‐positive peak counts per hpf (lymphocytes), and MCT‐positive peak counts per hpf (mast cells) were analyzed (within the esophageal epithelium). To assess epithelial‐mesenchymal transition (EMT), immunostaining for E‐Cadherin and Vimentin was performed as previously described [10]. For analysis, the number of vimentin positive cells per mm^2^ was counted in 10 different fields (mm^2^ hpf) per biopsy (with complete blinding to clinical and molecular findings). Immunostaining for LEKTI, a marker for epithelial integrity, was used to measure differences in epithelial barrier function. For analysis, the percentage of LEKTI expression was reported for 10 different fields (mm^2^ hpf) per biopsy (with complete blinding to clinical and molecular findings). For details on determined proteins and analyses see Supporting Information.

### 2.5 RNA Sequencing Studies

For this study, RNA sequencing data from 10 patients with non‐specific esophagitis were analyzed [2]. Active EoE (*n* = 10), GERD (*n* = 6), lymphocytic esophagitis (*n* = 5) and esophagus‐healthy individuals (*n* = 7) already included in our cohort and analyzed by RNA sequencing in our previous publication served as controls [2]. All RNA‐sequencing samples were derived from patients enrolled at the Swiss EoE Clinic. To minimize batch effects, all samples were processed concurrently and under identical conditions using a standardized protocol at a single genomic facility. RNA was extracted from FFPE samples using the automated Maxwell RSC RNA FFPE Kit on the Maxwell RSC Instrument (Promega, Dübendorf, Switzerland). Libraries for RNA‐seq were prepared with the SMARTer Stranded Total RNA‐Seq Kit v2—Pico Input Mammalian (Takara Bio USA Inc., CA, USA) and sequenced on an Illumina NovaSeq S1 flow cell (2 × 50 bp), following the protocol previously described [1]. The reads underwent alignment to the Ensembl GRCh38.p14 reference genome via STAR (v2.7.0e). Alignment quality was assessed by parsing HISAT2 alignment summaries. The median overall alignment rate was 86.6% (range: 56.1%–89.8%; 36/38 samples > 75%). Library sizes after filtering ranged from 7.9 M to 47.2 M genic reads (median: 22.4 M), with no systematic differences across phenotype groups. During feature counting, featureCounts was applied with a minimum mapping quality threshold of 10. Quality control was additionally performed using FastQC (v0.11.5) and RSeQC (v2.6.4). Differential expression analysis was conducted using either the DESeq2 R package (v1.34.0). Principal component analyses were performed using the *plotPCA* function of the same package. Pathway enrichment analyses were done using the *enricher* function of the clusterProfiler R package (v4.2.2) with pathways imported in R using the *msigdbr* function of the msigdbr package (v7.5.1). xCell analyses were carried out using the dedicated R package. Raw and processed bulk RNA sequencing data are deposited in the Gene Expression Omnibus (GEO) database repository under the accession number: GSE148381.

### 2.6 Patient and Public Involvement

Patients and/or the public were not involved in the design, conduct, reporting, and/or dissemination plans of this research.

### 2.7 Statistical Analyses

For statistical analyses, GraphPad Prism software version 8.3.0 and R version 3.6.0 were used. Metric data are presented as medians with IQR. Categorical data are depicted as percentage of the group total. For comparisons between two continuous variables, two‐sample *t*‐test and Wilcoxon rank‐sum test were used depending on whether data were normally distributed or not (ANOVA and Kruskal‐Wallis test in case of more than 2 groups). Comparison between categorical data was performed by using Chi‐square test or Fisher's exact test, if sample size was less than 10. For the purposes of this study, a *p*‐value of < 0.05 was considered statistically significant.

## 3 Results

### 3.1 Patient Characteristics

We identified 19 non‐specific esophagitis patients (6 males, all Caucasian) with a median age of 48 years (IQR 31–59) and a median diagnostic confirmation time of 39 months (IQR 12–113). The patients were followed for a median of 20 months (IQR 10–41). Symptom severity was considerable (12/19 patients, 63.2% had repetitive bolus impactions qualifying for severe dysphagia), while endoscopic activity was only mild (median EREFS 0, IQR 0–2). Per inclusion criteria, none of the patients had concomitant GERD. For details, see Table 1. No patient was on anti‐eosinophil treatment at the time of diagnosis, including topical and systemic steroids, dietary restrictions or PPI treatment.

**TABLE 1:** Patient, disease and follow‐up characteristics of non‐specific esophagitis patients.

	Non‐specific esophagitis *n* = 19
*Patient characteristics*	
Female sex	13 (68.4%)
Age at onset, years, median (IQR)	46 (29–53)
Age at diagnosis, years, median (IQR)	48 (31–59)
Atopic comorbidities	7 (36.8%)
Ancestry	
Caucasian	19 (100%)
Family history for EoE	6 (31.6%)
Previous PPI treatment	12 (63.2%)
Diagnostic confirmation time, months, median (IQR)	39 (12–113)
*Baseline disease activity*	
Symptomatic disease	19 (100%)
Endoscopic activity, EREFS, median (IQR)	0 (0–2)
Histological activity, EoE‐HSS, median (IQR)	0 (0–8.3)
*Follow‐up*	
Time of follow‐up, months, median (IQR)	20 (10–41)
Development of EoE	1 (5.3%)
Development of tissue eosinophilia (not meeting criteria for EoE)	3 (15.8%)
Steroid treatment	14 (73.7%)
Symptomatic response	12 (85.7%)
Endoscopic dilation	6 (31.6%)

### 3.2 Histological Assessment and Immunostaining

Non‐specific esophagitis diagnosis was made as described in the methods and schematically summarized in Figure 1A. No eosinophils/neutrophils were detected at baseline. EoE‐HSS was low (median grade/stage 0, 0–8.3). In fact, more than half of patients did not show any histological sign captured by the EoE‐HSS (10/19, 52.6%). Most frequently, spongiosis or basal zone hyperplasia were reported (in 8 patients). Immunostaining revealed detectable levels of lymphocytes in all patients (median peak lymphocyte count 15/hpf, IQR 9–24), but absent or very low numbers of eosinophils (median peak eosinophil count 1/hpf, IQR 0–2). In addition, only few mast cells were seen (median peak mast cell count 3/hpf, IQR 3–4). Of note, lymphocyte infiltrates were CRTH2 negative with a peak CRTH2+ cell count of 1 (IQR 0–1). Double positive CRTH2+/CD3+ cells were only seen in 3 patients. Number of mast cells, eosinophils and lymphocytes (peak per hpf) did not differ from esophagus healthy controls and GERD patients, while the number of eosinophils and mast cells was significantly lower compared to EoE. For details, see Table 2 and Figure S1 (for representative pictures) and Figure S2. Immunostaining for the protein Lympho‐Epithelial Kazal‐Type related Inhibitor (LEKTI), a marker for epithelial integrity, was significantly downregulated in patients with non‐specific esophagitis compared to healthy controls indicating an epithelial barrier defect (percentage of LEKTI positive area, median 16.5% vs. 25.4%, *p* = 0.008). See Figure 1B and Figure S1 (for representative pictures). Lamina propria tissue was available for conventional analysis in 14 patients, with subepithelial fibrosis only seen in 1 patient (7.1%). For 9 patients, immunostaining of the subepithelial tissue was performed, with assessment of two hpfs per patient. All 9 patients showed lymphocytes in the lamina propria (median peak lymphocyte count of 11, IQR 6–23), but no other cellular infiltrates, particularly no eosinophils.

**FIGURE 1:**
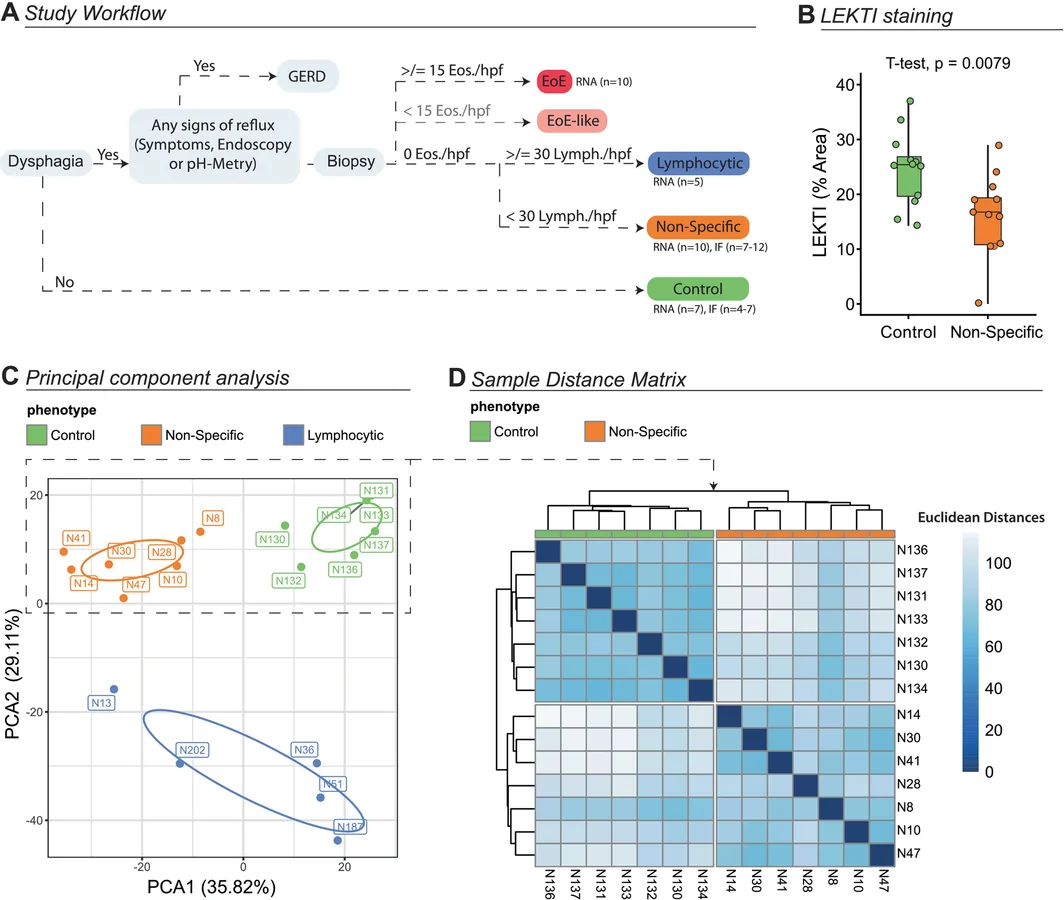
(A) Flow chart and classification of study patients according to the presence of symptoms, exclusion of GERD and H&E staining. (B) Boxplot showing the percentage area of esophageal tissue stained positive for LEKTI in non‐specific esophagitis versus healthy controls. Each point represents an individual patient. (C) PCA plot of healthy controls and patients with non‐specific esophagitis and lymphocytic esophagitis, with the exclusion of three outlier patients (for the complete PCA, see to Figure S1). (D) Matrix displaying the euclidean distances with unsupervised clustering of rows and columns between patients with non‐specific esophagitis and healthy controls, with the exclusion of three outlier patients with non‐specific esophagitis (for the complete matrix, see to Figure S1).

**TABLE 2:** Immunostaining performed in a subset of patients.

	Non‐specific esophagitis *n* = 12	Healthy controls *n* = 12	GERD *n* = 10	EoE *n* = 10
Peak eosinophil count/hpf, median (IQR)	1 (0–2)	1 (0–1)	1 (0–2)	36 (27–51)^a^
Peak lymphocyte count/hpf, median (IQR)	15 (9–24)	9 (9–10)	13 (9–23)	8 (8–15)
Peak mast cell count/hpf, median (IQR)	3 (3–4)	4 (3–6)	4 (3–5)	13 (11–15)^b^

*Note:* Cellular infiltration in esophageal biopsies obtained from non‐specific esophagitis patients compared to healthy controls, GERD, and EoE, based on immunostaining for EPX (eosinophils), CD3 (lymphocytes), and MCT (mast cells).

^a^ *p* < 0.0001 for EoE versus non‐specific esophagitis, EoE versus HC, EoE versus GERD.

^b^ *p* < 0.0001 for EoE versus non‐specific esophagitis, EoE versus HC, EoE versus GERD.

### 3.3 Follow‐Up

In the follow‐up, 14 patients were treated with topical steroids (symptomatic response in 12 of them, 85.7%). Six patients (31.6%) underwent endoscopic dilation for esophageal strictures. Examination of follow‐up biopsies revealed progression to EoE in one patient (5.3%). Esophageal eosinophilia below the cut‐off to diagnose EoE (< 15 eos/hpf) developed in three patients (15.8%).

### 3.4 RNA Sequencing Analyses

Bulk RNA sequencing was performed from esophageal biopsies at diagnosis of non‐specific esophagitis patients and compared to EoE, lymphocytic esophagitis, GERD, and esophagus healthy controls. Principal component (PC) analysis (PCA) of the transcriptomic data demonstrated clear separation of healthy controls from the disease groups (Figure S3A,B) with the exception of two non‐specific esophagitis patients (N42 and N37) clustering with healthy controls and one non‐specific esophagitis outlier clustering separately (N12). Euclidean distances in PC1/PC2 space were calculated from each sample to the centroid of the non‐specific esophagitis group, and the 99th percentile of this distribution was used as an outlier threshold. Sample N12 exceeded this cutoff and was therefore identified as a clear outlier and removed from further analysis (Figure S4). In contrast, samples N37 and N42 were positioned within the control centroid region, indicating a control‐like transcriptional profile; based on this misalignment, both were excluded from downstream analyses (Figure S4). The remaining 7 patients showed a clear clustering by phenotype according to PCA with confidence ellipse (Figure 1C), euclidean distance positioning in PC space (Figure S4), and unsupervised algorithm of the correlation matrix (Figure 1D) without any overlap with healthy controls. For details, also see Figure S3C–H. We compared the transcriptome to 6 GERD patients and denoted clear segregation of the two conditions (Figure S5).

We next performed a differential gene expression analysis between non‐specific esophagitis patients and healthy controls and identified 2152 positively differentially expressed genes (DEGs) according to an adjusted *p*‐value cutoff of < 0.05 and a logarithmic fold change of > 1 (Figure 2A and Table S1). Pathway enrichment analysis of these DEGs using the Hallmarks gene set of the Msigdb database was run to delineate gross biological processes [11, 12]. Among those, epithelial‐mesenchymal transition, myogenesis, and KRAS signaling up were the most significantly upregulated hallmark pathways; see Figure 2B and Table S2. To investigate these processes in more detail, pathway analyses were repeated using the Reactome pathways gene sets [13] and the resulting pathways were grouped into four biological categories (tissue remodeling, muscle contraction, fibrosis, and immune‐related pathways) by unsupervised hierarchical clustering (Figure 2C and Table S3). This approach revealed a highly enriched remodeling process including extracellular matrix organization and degradation of the extracellular matrix, but also fibrosis‐related pathways such as collagen formation, collagen degradation and collagen chain trimerization. While immune pathways were also enriched, with leishmania and parasite infection pointing towards a Th2 process, enrichment of classical EoE‐related pathways was not apparent, such as involvement of IL4, IL5, IL13, CCL26, or TSLP (Figures S6 and S7).

**FIGURE 2:**
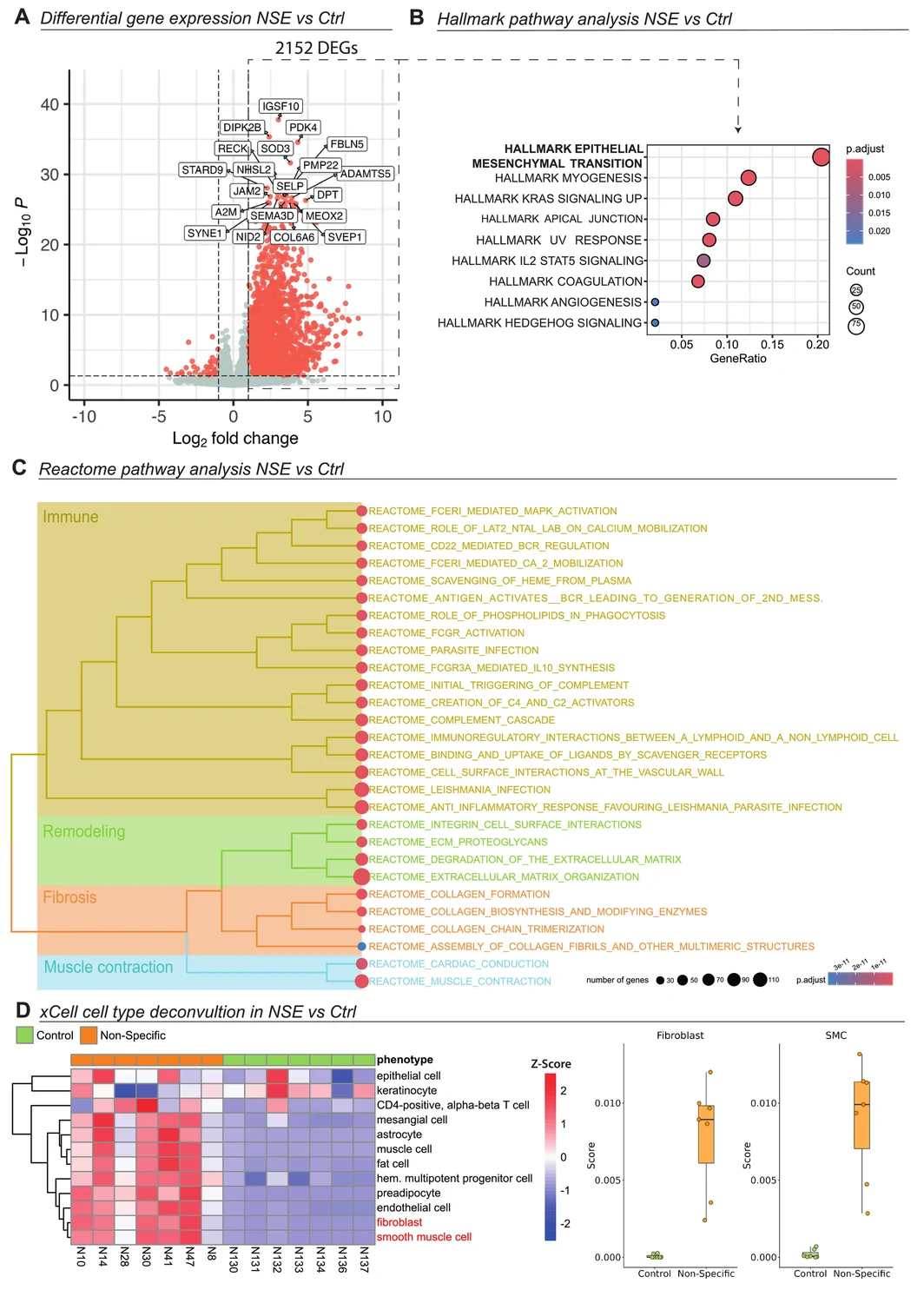
(A) Volcano plot displaying the DEGs between non‐specific esophagitis and healthy controls as determined by DEseq2 differential expression analysis. Vertical and horizontal dashed lines show logarithmic fold change cut off of 1 and the adjusted *p*‐value cut‐off of 0.05. Genes colored in red are considered statistically significant. In total 2152 positively upregulated DEGs were found and the top 20 gene names are displayed. (B) Bubble plot displaying the results of the pathway enrichment analysis performed on the 2152 positively DEGs in non‐specific esophagitis compared to healthy controls using the Hallmark gene sets from the MSigDB database. Pathways are ordered by the ratio of DEGs within each gene set to the total genes in the set. (C) Tree plot displaying the results of the pathway enrichment analysis performed on the 2152 positively DEGs in non‐specific esophagitis compared to healthy controls using the Reactome gene sets from the MSigDB database. Unsupervised clustering ordered the pathways based on the common genes composing them, cluster number was arbitrarily set to 4. Pathways are colored based on their clustering and a global biological process was manually assigned to each of the 4 clusters. (D) Heatmap with unsupervised clustering of rows displaying the results of the xCell2 analysis performed on the count matrix of non‐specific esophagitis and healthy control samples. Boxplots on the right show fibroblast and smooth muscle cell scores for non‐specific esophagitis versus healthy controls. NSE, non‐specific esophagitis; Ctrl, healthy controls.

To discern enriched cell types, xCell analysis [14] was performed based on the 2152 differentially expressed genes. Thereby, an enrichment of fibroblasts, but not inflammatory cells, was detected, paralleling fibrotic activity and tissue remodeling seen in the pathway enrichment analyses. For details, see Figure 2D.

We next compared the non‐specific esophagitis transcriptome to that of patients with EoE, lymphocytic esophagitis, and esophagus healthy controls to identify genes that were significantly differentially expressed consistently across all comparisons. We identified a total of 95 genes that were significantly up‐ (51) or downregulated (44) in non‐specific esophagitis (Tables S4 and S5). Hierarchical clustering based on the expression of these 95 genes effectively distinguished non‐specific esophagitis patients from the other groups (Figure 3A). Among the 51 upregulated genes, several are known to play key roles in the regulation of fibrosis, tissue elasticity, and inflammation, including FBLN5, RECK, SDC2, ADAMTSL1, TGFBR3, PDGFC, PIK3R1, and FMOD (Figure 3B). Similarly, several of the 44 downregulated genes are involved in the regulation of tissue remodeling and fibrosis, such as SCIRT, CXADR, GCH1, and PLAU (Figure 3C). The top upregulated gene IGSF10 is known to be upregulated in patients with lung fibrosis and systemic sclerosis.

**FIGURE 3:**
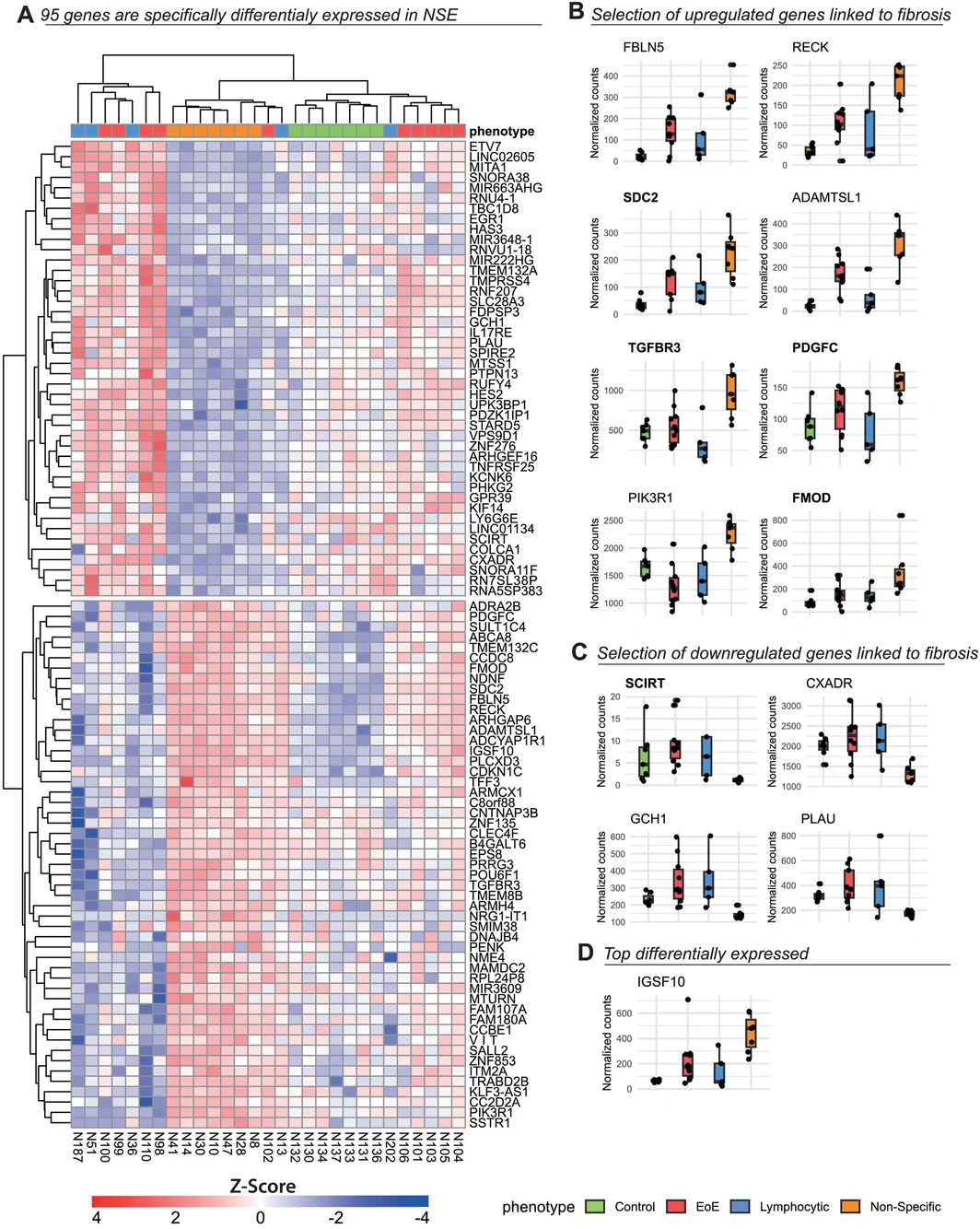
(A) Heatmap with clustering of rows (genes) displaying the expression of the 95 genes significantly and consistently up‐ or downregulated in non‐specific esophagitis patients vs. all other groups (healthy controls, lymphocytic esophagitis and EoE). Red color indicates upregulation, blue color indicates downregulation. (B) Boxplots (normalized counts) of key selected upregulated genes from (A) linked to fibrosis and tissue remodeling. (C) Boxplots (normalized counts) of key selected downregulated genes from (A) linked to fibrosis and tissue remodeling. (D) Boxplot (normalized counts) displaying the expression of IGSF10, the most significantly upregulated gene in non‐specific esophagitis. NSE, non‐specific esophagitis.

To exclude confounding by age and sex, we re‐ran the differential expression analysis including age and sex as covariates in the DESeq2 model (~phenotype + age + sex). Log2 fold changes between the adjusted and unadjusted models were highly correlated (Pearson *r* = 0.934), and 89.5% of the top 1500 differentially expressed genes overlapped between both analyses, demonstrating that the transcriptomic signature is largely independent of age and sex.

### 3.5 Assessment of Epithelial‐Mesenchymal Transition

Given the involvement of extracellular matrix remodeling and fibrosis in non‐specific esophagitis, we next assessed EMT in a subset of our patients (*n* = 10). EMT is a key step in early fibrosis as epithelial cells with a mesenchymal phenotype behave like fibroblasts [15]. Immunofluorescence for E‐cadherin and Vimentin has been used as a surrogate marker for EMT and fibrosis [10]. In addition, EMT has been previously shown to correlate with disease severity in EoE and indirectly with esophageal distensibility [16, 17]. Immunofluorescent histological staining showed high number of Vimentin positive cells (per mm^2^ hpf) in EoE, denoting a clear EMT process, and a low number in control biopsies. Biopsies from patients with non‐specific esophagitis displayed intermediate levels (Figure 4A,B). These results indicate an ongoing EMT in non‐specific esophagitis albeit at levels lower than in EoE.

**FIGURE 4:**
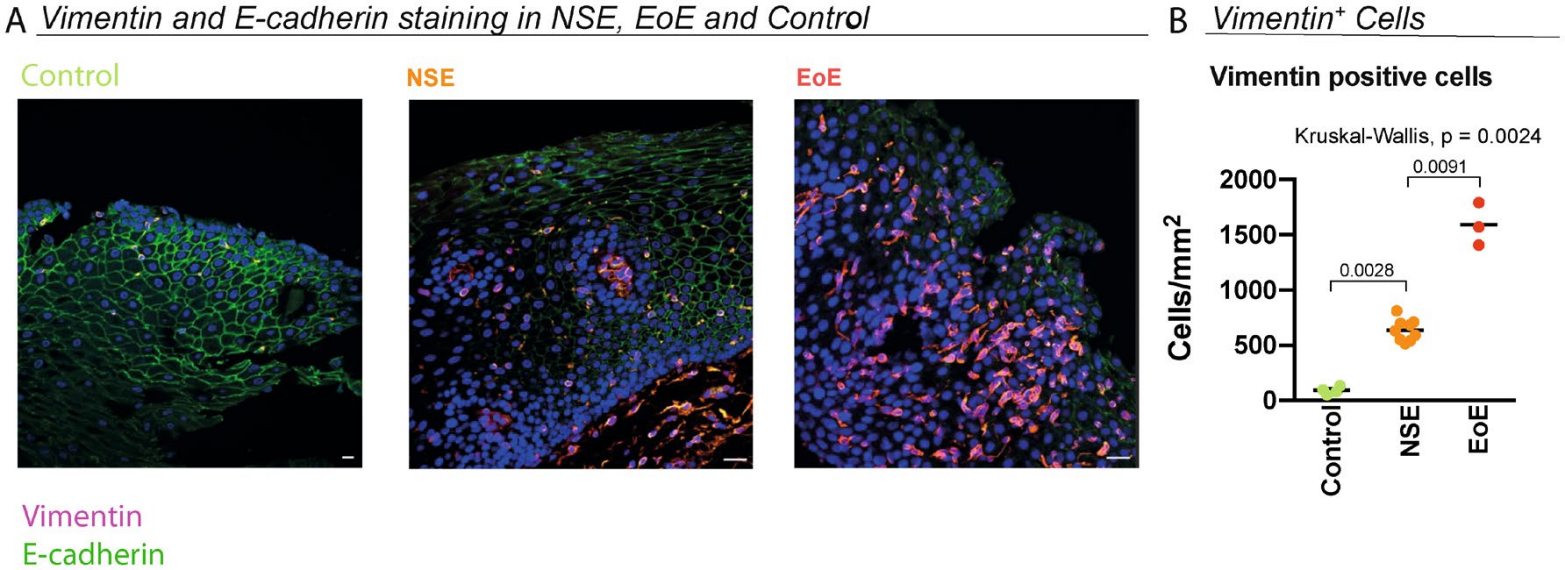
(A) Esophageal expression of Vimentin (purple) and E‐cadherin (green) in healthy controls, non‐specific esophagitis (NSE) patients, and EoE patients. Hoechst H3570 was used for nuclear staining. (B) Quantification of vimentin staining (Vimentin positive cells per mm^2^). NSE, non‐specific esophagitis; EoE, eosinophilic esophagitis.

## 4 Discussion

Non‐specific esophagitis has been recently described in patients with symptoms compatible with EoE, but not fulfilling the criteria for EoE or lymphocytic esophagitis [2]. Here, we present an in‐depth analysis of baseline and follow‐up data of 19 non‐specific esophagitis patients over 20 months. Our data suggest the presence of a non‐eosinophilic disease phenotype with fibrotic traits that shows esophageal dysfunction symptomatically, but is clearly distinct pathologically and molecularly. Our main findings are: (1) Despite the absence of eosinophilic infiltration, most patients with non‐specific esophagitis present with severe dysphagia; (2) Decreased LEKTI expression suggests an underlying epithelial barrier dysfunction; (3) RNA sequencing revealed several pro‐fibrotic traits and a clearly distinct molecular profile compared to EoE, lymphocytic esophagitis, GERD, and healthy controls; and (4) EMT was significantly increased compared to healthy controls.

Non‐specific esophagitis patients show considerable symptom burden. All adult patients presented with moderate dysphagia and five of them experienced food bolus impaction (severe dysphagia). The severe clinical presentation contrasts the rather mild endoscopic disease severity. In fact, the majority of patients showed a completely normal esophagus on endoscopic assessment, which clearly separates non‐specific esophagitis from EoE, where endoscopic abnormalities are detected in 90%. Moreover, the majority of patients do not show EoE typical histological changes beyond eosinophilic infiltration. In addition, these patients lack of subepithelial eosinophilia. Thus, non‐specific esophagitis is not a subepithelial form of EoE. Our data are compatible with the hypothesis that fibrosis and epithelial barrier dysfunction contribute to the disease pathogenesis.

Minimal cellular infiltration was confirmed by immunostaining of non‐specific esophagitis biopsies. Eosinophil infiltration was absent, and only very few mast cells were detected. Lymphocytes were CRTH2 negative, which again shows a clear distinction compared to EoE, where lymphocytes are CRTH2 positive [18]. In addition, the non‐specific distribution and the non‐significant increase compared to healthy controls question the pathogenic role of lymphocytes (and inflammation) in non‐specific esophagitis. It is intriguing that lymphocyte counts were not different from GERD and healthy controls based on immunostaining. Currently, non‐specific esophagitis is defined by H&E staining taking into account lymphocyte counts and other histological changes beyond cellular infiltrates. The latter appear more important than lymphocyte counts. On the other hand, lymphocyte counts are probably underreported in H&E staining from control patients as they are considered normal. A decreased LEKTI expression suggests an underlying epithelial barrier dysfunction. The epithelial barrier hypothesis has gained increasing attention in the pathogenesis of esophageal diseases [19, 20, 21]. The looming question is whether disruption of the epithelial barrier is an early disease mechanism enabling antigen crossing or rather a bystander effect of the inflammatory processes [22]. The finding of an epithelial barrier defect in the context of absent inflammation in our study—however—is compatible with the hypothesis that epithelial barrier dysfunction is indeed a contributing pathogenic factor, although more in‐depth analyses of epithelial barrier (beyond LEKTI) are needed.

Non‐specific esophagitis shows a unique mRNA profile, with a particular involvement of fibrosis. Despite the above‐mentioned lack of inflammation, the transcriptomic profile of non‐specific esophagitis is clearly distinct from healthy controls and GERD, and also from lymphocytic esophagitis and active EoE. More importantly, pathway analyses suggest the involvement of fibrogenesis, tissue remodeling, and EMT, with a particular role for fibroblasts. The identified up‐ and downregulated genes with involvement in extracellular matrix and fibrosis development are transcriptomic associations and need to be analyzed in further studies involving more patients. Our hypothesis‐generating findings contrast, however, with the low rates of fibrotic changes seen on endoscopy and the negligible proportion of patients with subepithelial fibrosis. Thus, the most widely used tools in EoE management—endoscopy and histological analyses—do not appear to be sufficient to assess the presence of fibrosis in non‐specific esophagitis [23].

Presence of fibrotic changes was confirmed by EMT analysis in our study. EMT is one of the key mechanisms of fibrosis in EoE, where epithelial cells take on characteristics of mesenchymal cells including collagen production, migration, and contraction [7]. While EMT has been demonstrated to be an early fibrotic step in EoE, we show in our study that EMT appears to occur even in the absence of inflammation or with only low‐grade inflammation. This supports the hypothesis that fibrosis can develop with minimal or only non‐classical inflammation and does not appear to rely on a considerable tissue eosinophilia. Whether or not this represents a burned‐out inflammatory process remains an open question. However, the fact that at least one of our patients progressed to EoE argues against such a burned‐out status.

Our study has several strengths and limitations. It is the first study that systematically describes patients with non‐specific esophagitis, with a detailed analysis using expert histological review, immunostaining (including markers for EMT and epithelial barrier function) and RNA sequencing (with detailed pathway enrichment analyses). Thereby, it supports the concept of a distinct fibrotic phenotype of the esophagus beyond the initial description as a potential EoE variant. Limitations are as follows: First, GERD was not systematically excluded by using pH‐metry. However, our approach of excluding GERD has been previously used in several studies and is often used in clinical practice [2]. Second, EndoFLIP data are missing. EndoFLIP is an increasingly used tool to assess esophageal distensibility. In fact, association with fibrotic changes has been described in EoE patients [24]. It would have been of particular interest to investigate esophageal distensibility in our patients and to clinically and functionally validate the EMT data. However, these patients initially presented at most of our centers before the availability of EndoFLIP. Third, the sample size was small, but we were still able to discern key mechanistic differences when compared to EoE, lymphocytic esophagitis, and GERD. Fourth, conclusions about epithelial barrier function are inferred from decreased LEKTI expression. Finally, high rates of steroid treatment may have masked the true natural history of the disease, including its potential for progression. Taken together, we report on a hypothesis‐generating study, while for a definitive description of a new entity, more data, particularly longer follow‐up is needed. Potential diagnostic and therapeutic implications are as follows: To assess fibrotic features and esophageal diseases, it is not sufficient to only focus on inflammatory changes. In particular, it is not sufficient to exclude a disease solely by the fact that eosinophilic infiltration is absent. From a therapeutic standpoint, future studies should assess fibrotic changes in addition to pure inflammatory readouts. Here, the novel diagnostic tool EndoFLIP should close an important gap and has already been implemented in ongoing studies of esophageal diseases.

In conclusion, non‐specific esophagitis appears to be a distinct fibrotic phenotype of the esophagus, without any considerable overlap with GERD and other chronic inflammatory disorders such as EoE or lymphocytic esophagitis. Furthermore, our study highlights the mismatch between assessment of fibrosis by RNA sequencing and EMT compared to conventional histology and endoscopy. Our data suggest development of fibrotic traits independently of inflammatory changes, in particular tissue eosinophilia.

## Author Contributions

Study concept and design: M.P., T.G., A.S., and H.‐U.S.; acquisition of data: T.G., A.S., L.B., M.H.C., M.C., E.S.D., G.T.F., I.H., N.G., I.H., F.J.M., and A.M.S.; histological examination: M.H.C.; analyses and interpretation of data: M.P., E.G., M.W., T.G., A.S., D. Simon, D. Stojkov, M.H.C., and H.‐U.S.; drafting of manuscript: M.P., T.G., A.S., and H.‐U.S.; critical revision of the manuscript for intellectual content: D. Stojkov, D. Simon, E.G., M.W., M.H.C., M.C., E.S.D., G.T.F., N.G., F.J.M., L.B., and A.M.S.; supervision: T.G., A.S., and H.‐U.S.

## Funding

This work was supported by grants from the Swiss National Science Foundation to HUS (grant no. 310030_184816), AMS (grant no. 32003B_204751/1), AS (grant no. 32003B_160115) and TG (grant no. P2ZHP3_168561), a young investigator award from the Swiss Society of Gastroenterology to TG, research grants from the Novartis Foundation for Medical‐Biological Research to TG and HUS, a research award from the Swiss IBDnet to TG, the FAN academic excellence grant from the University of Zurich to TG, and a training grant from the Consortium of Eosinophilic Gastrointestinal Disease Researchers (CEGIR) to TG. CEGIR (U54 AI117804) is part of the Rare Disease Clinical Research Network (RDCRN), an initiative of the Office of Rare Diseases Research (ORDR), National Center for Advancing Translational Sciences (NCATS), and is funded through collaboration between the National Institute of Allergy and Infectious Diseases (NIAID), National Institute of Diabetes and Digestive and Kidney Diseases (NIDDK), and NCATS. CEGIR is also supported by patient advocacy groups including the American Partnership for Eosinophilic Disorders (APFED), Campaign Urging Research for Eosinophilic Disease (CURED) and Eosinophilic Family Coalition (EFC). The study was further supported by a research grant from the Swiss EoE Foundation to HUS (grant no. 2021‐03) and TG (grant no. 2023‐01).

## Disclosure

*Guarantors of the Article*: M.P. M.D. Ph.D., A.S. M.D., H.‐U.S. M.D., Ph.D. and T.G. M.D.

*RNA‐Seq*: RNA‐seq was performed by the Next Generation Sequencing (GS) Platform of the University of Bern. RNA‐seq data has been deposited in the Gene Expression Omnibus (GEO), accession number **GSE148381**.

## Conflicts of Interest

T.G. has consulting contracts with Sanofi‐Regeneron, Janssen, BMS, Takeda, Abbvie and Falk Pharma GmbH, received travel grants from Falk Pharma GmbH and Vifor, speaker's fee from Norgine and an unrestricted research grant from Novartis. A.S. has consulting contracts with Actelion, Celgene‐Receptos, Falk Pharma GmbH, Roche‐Genentech, GSK, Novartis, Nutricia and Sanofi‐Regeneron. A.M.S. is a consultant for Falk Pharma GmbH, Adare Pharmaceuticals Inc., Celgene‐Receptos, and Sanofi‐Regeneron. M.H.C. is a consultant for Astra Zeneca, Allakos, Arena, Celgene, Esocap, GlaxoSmithKline, Regeneron, Sanofi, and Shire. e. L.B. reports fees for consulting/advisory board from Abbvie, Amgen, BMS, MSD, Vifor, Falk, Esocap, Janssen, Calypso, Ferring, Pfizer, Takeda, Janssen, Sanofi. G.T.F. is a consultant for Takeda, has received research support from Holoclara and Arena/Pfizer and is Chief Medical Officer of EnteroTrack. M.C. has received consulting fees from Regeneron, Adare/Ellodi, AstraZeneca, Sanofi, Bristol Myers Squibb, Allakos, Shire/Takeda, Phathom, Recludix, Uniquity Bio, and has received research funding from Regeneron, Allakos, Shire/Takeda, AstraZeneca, Celgene, Adare/Ellodi, Danone. E.S.D. has received research funding from Adare/Ellodi, Allakos, Arena/Pfizer, AstraZeneca, Celldex, Eupraxia, Ferring, GSK, Meritage, Miraca, Nutricia, Celgene/Receptos/BMS, Regeneron, Revolo, Sanofi, Shire/Takeda and Uniquity, has received consulting fees from Abbvie, Adare/Ellodi, Akesobio, Alfasigma, ALK, Allakos, Amgen, Apogee, Apollo, Aqilion, Arena/Pfizer, Aslan, AstraZeneca, Avir, Biocryst, Bryn, Calypso, Celgene/Receptos/BMS, Celldex, EsoCap, Eupraxia, Dr. Falk Pharma, Ferring, GI Reviewers, GSK, Holoclara, Invea, Knightpoint, LucidDx, Morphic, Nexstone Immunology/Uniquity, Nutricia, Parexel/Calyx, Phathom, Regeneron, Revolo, Robarts/Alimentiv, Sanofi, Shire/Takeda, Target RWE, Third Harmonic Bio, Upstream Bio and has received Allakos, Aqilion, Holoclara and Invea. F.J.M. is a consultant for Takeda, Regeneron, and Sanofi. H.‐U.S. is a consultant for AstraZeneca, GlaxoSmithKline, and Esocap. The other authors have no competing interests to declare. No company representative was involved in conception, writing, or financing of this study.

## Supporting information


**Table S1:** List of 2152 positively differentially expressed genes in patients with non‐specific esophagitis vs. healthy controls.

**Table S2:** Hallmark pathway analysis for differentially expressed genes in non‐specific esophagitis patients versus healthy controls.

**Table S3:** Reactome pathways analysis for differentially expressed genes in non‐specific esophagitis patients vs. healthy controls.

**Table S4:** List of 51 upregulated genes in non‐specific esophagitis patients compared to EoE, lymphocytic esophagitis and healthy controls.

**Table S5:** List of 44 downregulated genes in non‐specific esophagitis patients compared to EoE, lymphocytic esophagitis and healthy controls.

**Figure S1:** Representative immunostaining pictures for CD3/EPX, MCT and LEKTI.
**Figure S2:** Quantification of CRTH2 positive cells per mm2 for healthy controls vs. non‐specific esophagitis vs. EoE.
**Figure S3:** (A) PCA plot of healthy controls and patients with EoE, non‐specific esophagitis and lymphocytic esophagitis. (B) Matrix displaying the euclidean distances with unsupervised clustering of rows and columns between patients with non‐specific esophagitis, EoE, lymphocytic esophagitis and healthy controls. (C) PCA plot of healthy controls and patients with EoE, non‐specific esophagitis and lymphocytic esophagitis, with the exclusion of three outlier patients (N12, N37 and N42). (D) Matrix displaying the euclidean distances with unsupervised clustering of rows and columns between patients with non‐specific esophagitis, EoE, lymphocytic esophagitis and healthy controls, with the exclusion of three outlier NSE patients (N12, N37 and N42). (E) PCA plot of healthy controls and patients with non‐specific esophagitis and lymphocytic esophagitis, with the exclusion of three outlier NSE patients (N12, N37 and N42). (F) Matrix displaying the euclidean distances with unsupervised clustering of rows and columns between patients with non‐specific esophagitis and lymphocytic esophagitis and healthy controls, with the exclusion of three outlier NSE patients (N12, N37 and N42). (G) PCA plot of healthy controls and patients with non‐specific esophagitis, with the exclusion of three outlier NSE patients (N12, N37 and N42). (H) Matrix displaying the euclidean distances with unsupervised clustering of rows and columns between patients with non‐specific esophagitis and healthy controls, with the exclusion of three outlier NSE patients (N12, N37 and N42).
**Figure S4:** Principal component analysis (PC1–PC2 using the top 500 most variable genes) was used to compute Euclidean distances from each Non‐Specific Esophagitis (NS) biopsy to the NS centroid (DNS) and the Control centroid (DCTRL). Samples positioned below the diagonal line (DCTRL<DNS) are more transcriptionally similar to Controls (“Control‐like”), while samples above the line classify as “NS‐like”. The vertical dotted line denotes the 99th percentile of the NS distance distribution, used as a robust cutoff to flag atypical samples. Patient N12, highlighted in red with a black outline, lies beyond this threshold and is therefore identified as an extreme outlier relative to the NS cohort.
**Figure S5:** PCA plot of healthy control and patients with GERD, non‐specific esophagitis and lymphocytic esophagitis, with the exclusion of three outlier NSE patients (N12, N37 and N42).
**Figure S6:** Bubble plot displaying the complete results of the pathway enrichment analysis performed on the 2152 positively DEGs in non‐specific esophagitis compared to healthy controls using the Reactome gene sets. Pathways are ordered by the ratio of DEGs within each gene set to the total number of genes in the set.
**Figure S7:** Heatplot displaying the genes and their expression in Reactome pathways enriched in non‐specific esophagitis compared to healthy controls. Pathways are color‐coded according to their clustering in the tree plot of Figure 3C.

## Data Availability

The data that support the findings of this study are available on request from the corresponding author. The data are not publicly available due to privacy or ethical restrictions.
