# Supplementary material for: Molecular Characterization of Non‐Specific Esophagitis Reveals a Distinct Non‐Eosinophilic Phenotype With Fibrotic Traits
Source: Allergy. 2026 May 1;81(10):3620–30. doi: 10.1111/all.70364 (PMC13646858; doi:10.1111/all.70364)
Supplement: Supplementary file 1 [file ALL-81-3620-s005.docx]

SUPPLEMENTARY MATERIAL

Title: Molecular characterization of non-specific esophagitis reveals a novel non-eosinophilic fibrotic esophageal disease

List of authors: Marc Pfefferle MD PhD^1,10,14^, Darko Stojkov PhD^2^, Dagmar Simon MD^3^, Emilie Gueguen MSc^1^, Marcin Wawrzyniak PhD^1^, Margaret H. Collins MD^4^, Mirna Chehade MD MPH^5^, Evan S. Dellon MD MPH^6^, Glenn T. Furuta MD^7^, Ikuo Hirano MD^8^, Nirmala Gonsalves MD^8^, Fouad J. Moawad MD^9^, Luc Biedermann MD^1^, Alain M. Schoepfer MD^10^, Alex Straumann MD^1^, Hans-Uwe Simon MD PhD^3,11+^, and Thomas Greuter MD^1,10,12+^

Author affiliations: ^1^Department of Gastroenterology and Hepatology, University Hospital Zurich, Zurich, Switzerland, ^2^Institute of Pharmacology, University of Bern, Bern, Switzerland, ^3^Department of Dermatology, Inselspital, Bern University Hospital, University of Bern, Bern, Switzerland, ^4^Division of Pathology, Cincinnati Children`s Hospital Medical Center, Department of Pathology, University of Cincinnati College of Medicine, Cincinnati, Ohio, ^5^Mount Sinai Center for Eosinophilic Disorders, Icahn School of Medicine at Mount Sinai, New York, New York, ^6^Center for Esophageal Diseases and Swallowing, Division of Gastroenterology and Hepatology, University of North Carolina School of Medicine, Chapel Hill, North Carolina, ^7^Department of Pediatrics, Gastrointestinal Eosinophilic Diseases Program, University of Colorado School of Medicine, Digestive Health Institute, Children’s Hospital Colorado, Aurora, Colorado, ^8^Division of Gastroenterology and Hepatology, Northwestern University, Chicago, Illinois, ^9^Division of Gastroenterology, Scripps Clinic, La Jolla, California, ^10^Division of Gastroenterology and Hepatology, University Hospital Lausanne – Centre Hopitalier Universitaire Vaudois (CHUV), Lausanne, Switzerland, ^11^Institute of Biochemistry, Brandenburg Medical School, Neuruppin, Germany, ^12^Division of Gastroenterology and Hepatology, EOC Lugano, Lugano, Switzerland, ^13^Faculty of Biomedical Sciences, Università della Svizzera italiana, Lugano Switzerland, ^14^Department of Internal Medicine, Limmattalspital, Schlieren, Switzerland

**TABLE OF CONTENTS**

1. Supplementary Methods

2. Supplementary Figures

3. Supplementary Tables

4. References

**1. Supplementary Methods**

*Study design*

This was an observational multicenter study involving adult patients with non-specific esophagitis, included in the EoE variant database. This database comprises of patients from 6 EoE referral centers in Switzerland (n=1) and the United States (n=5). These centers are: Swiss EoE Clinic, Mount Sinai Center for Eosinophilic Disorders, Center for Esophageal Diseases and Swallowing at UNC Chapel Hill, Gastrointestinal Eosinophilic Diseases Program at Children’s Hospital Colorado, Northwestern Eosinophilic Digestive Disease Program at Northwestern University, and Scripps Clinic in La Jolla. For the purpose of the current study, we analyzed clinical, endoscopic, histological, immunohistochemical and molecular data of patients with non-specific esophagitis. Healthy individuals, EoE patients, patients with lymphocytic esophagitis and gastro-esophageal reflux disease (GERD) patients also included in the database served as controls. Healthy controls underwent upper endoscopy for a non-esophageal cause (eg dyspepsia).The study was approved by the local ethics committee of each of the participating centers (EKNZ 2015-388/CER-VD 148-15, COMIRB 07-0888, UNC IRB 14-1442, IRB-16-6910, STU00094108, ISMMS HS-10-00070).

*Patients and data collection*

The EoE variant database includes patients presenting with symptoms that are compatible with EoE, but absence of esophageal eosinophilia defined by a cutoff of 15 eosinophils per hpf (in the absence of anti-eosinophil treatment or dietary restrictions and in the absence of GERD). GERD was excluded as previously described: clinically by lack of typical symptoms, endoscopically by absence of hiatal hernia or signs of reflux esophagitis (including a Schatzki ring), by lack of proton-pump inhibitor (PPI) response assessed histologically after at least 8 weeks of treatment and/or by negative pH-monitoring. (1) Esophageal biopsies were all re-examined by an EoE reference pathologist. For the purpose of this study, only patients classified as having non-specific esophagitis were analyzed.

The following definitions were applied:

| Non-specific esophagitis | Histological infiltration of lymphocytes not fulfilling the numerical and distributional criteria of lymphocytic esophagitis, in the absence of tissue eosinophilia, corresponding to a low-grade inflammation. |
| --- | --- |
| Lymphocytic esophagitis | Lymphocyte-predominant inflammation with high numbers of intraepithelial lymphocytes (≥30 per hpf), gathered mainly in peripapillary fields, peripapillary spongiosis (dilated intercellular spaces) and absence of intraepithelial granulocytes |
| Eosinophilic esophagitis | Eosinophil-predominant inflammation with high numbers of intraepithelial eosinophils (≥15 per hpf), in the absence of differential diagnoses explaining tissue eosinophilia. |

The structured data collection was performed by means of a standardized spreadsheet. All data were anonymized. The following data were collected: patient demographics, disease characteristics including clinical, endoscopic and histological EoE presentation. EoE Endoscopic Reference Score (EREFS) grading and classification system was used to classify endoscopic disease activity based on the available endoscopic pictures to grade endoscopic disease activity.(2) The EoE Histological Scoring System EoE-HSS was used for grading and staging of histological disease activity as assessed by an EoE reference pathologist. (3)

*Immunostaining and confocal microscopy*

Our immunostaining protocol has been previously described. (1) Paraffin-embedded human tissue slides were deparaffinized and rehydrated with graded ethanol dilutions. After antigen retrieval in a pressure cooker using EDTA-Tris buffer for EPX, CD3, and CRTH2 (10 mM Tris Base, 1 mM EDTA, pH 9.0, with 0.01% saponin) or Citrate buffer for E-Cadherin and Vimentin staining (10 mM sodium citrate, 0.01% saponin, pH 6.0), non-specific binding was prevented by preincubation of tissue sections with a blocking buffer (PBS with 0.01% saponin containing human immunoglobulins, normal goat serum, and 7.5% BSA) at room temperature (RT) for 1 h. Indirect immunofluorescence staining was performed by incubating the paraffin sections in blocking solution, at 4°C, overnight with the following primary antibodies: Mouse monoclonal anti-human E-Cadherin (clone # HECD-1) and rabbit monoclonal anti-human Vimentin (clone EPR3776), purchased from Abcam (Cambridge, UK). Mouse monoclonal anti-human EPX (clone MM25-82 2) was obtained from Lee Laboratories, Mayo Clinic, Rochester, USA. Mouse monoclonal anti-human mast cell tryptase (MCT) (clone AA1) and anti-human CD3 (clone F7.2.38) were both from Dako, distributed by Agilent (Santa Clara, USA). Rabbit polyclonal anti-human LEKTI (SPINK5) was purchased from Novus Biologicals (Littleton, USA). Mouse monoclonal anti-human prostaglandin D2 receptor (DP2 / CRTH2) was from Santa Cruz Biotechnology, Inc, Heidelberg, Germany. After several washes in PBS plus 0.01% saponin, secondary Alexa Fluor 488–conjugated goat anti-mouse (1:400) and Alexa Fluor 532–conjugated goat anti-rabbit (1:400) antibodies (ThermoFisher Scientific, Basel, Switzerland) were applied and tissue samples incubated at RT for 1 h. Samples were washed and nuclear DNA was stained with Hoechst 33342 for an additional 10 min. Samples were then mounted in Prolong Gold mounting medium (ThermoFisher Scientific) and image acquisition was performed using confocal laser scanning microscopy LSM 800 (Carl Zeiss Micro Imaging) with a 20x or 40x/1.40 Oil DIC objective and analyzed with IMARIS software (Oxford Instruments, Zürich, Switzerland). For better visualization, the gamma correction function, together with min/max thresholds of Imaris software, were used to optimize the image display by intensifying the grey values. For overall qualitative analysis, stained tissues were scanned using an automated slide scanner (3DHISTECH), with Quant Centre software, using Cell Count module (Sysmex Suisse Horgen, Switzerland).

*RNA Isolation and RNA sequencing studies*

For the purpose of this study, FFPE esophageal biopsy tissue blocks were used (10 non-specific esophagitis patients, 6 patients with GERD, 10 patients with EoE, 5 patients with lymphocytic esophagitis, 7 healthy controls). Our RNA isolation (using Maxwell® RSC RNA FFPE Kit in Maxwell® RSC Instrument, Promega, Dübendorf, Switzerland) and RNA sequencing protocols (using SMARTer Stranded Total RNA-Seq Kit v2 - Pico Input Mammalian, Takara Bio USA, Inc., CA, USA and NovaSeq S1 flow cell (2 x 50 bp), Illumina, CA, USA) have been previously described. (1) The quality of the RNA-seq data was assessed using fastqc v. 0.11.5 (4) and RSeQC v. 2.6.4. (5) The reads were mapped to the reference genome using HiSat2 v. 2.1.0. (6) FeatureCounts v. 1.6.0 was used to count the number of reads overlapping with each gene as specified in the genome annotation (Homo_sapiens.GRCh38.94). (7)

Raw counts were normalized using the size factor method implemented in the DESeq2 package (v1.46.0). DEGs were identified using DESeq2 with an adjusted p-value threshold (Benjamini-Hochberg correction) of padj ≤ 0.05 and |log2FC| ≥ 1. (8) PCA were computed using *plotPCA()* function of the DESeq2 package (top 500 variable genes) and plotted using the ggplot2 package’s (v3.5.1) *geom_point()* and ggpubr (0.6.0) package’s *stat_conf_ellipse(level = 0.95)* function. Sample distance matrices were generated using the *dist()* function of the stats (v3.6.2) package. Differential gene expression was performed between all patient groups using the *DESeq()* function of the DESeq2 package. Ensembl gene IDs were converted to HNGC symbols with the AnnotationDbi (v1.68.0), org.Hs.eg.db (v3.20.0) and biomaRt (v2.62.1) packages.

Pathway enrichment was performed using over-representation analysis (ORA) in clusterProfiler (v4.16.0), using the detected DEGs as input and all expressed genes as background. Default clusterProfiler settings were applied (Benjamini-Hochberg correction, p ≤ 0.05, q ≤ 0.20) (<https://www.liebertpub.com/doi/10.1089/omi.2011.0118>) was used for pathway enrichment analysis on the significant genes. The enrichplot R package version 1.28.2 (<https://bioconductor.org/packages/enrichplot>) was used for pathway enrichment result visualization. Raw counts are available in the Gene Expression Omnibus (GEO), accession number GSE148381.

**2. Supplementary Figures**

**Supplementary figure 1:** Representative immunostaining pictures for CD3/EPX, MCT and LEKTI.

**Supplementary figure 2:** Quantification of CRTH2 positive cells per mm2 for healthy controls vs non-specific esophagitis vs EoE.

**Supplementary figure 3:** A) PCA plot of healthy controls and patients with EoE, non-specific esophagitis and lymphocytic esophagitis. B) Matrix displaying the euclidean distances with unsupervised clustering of rows and columns between patients with non-specific esophagitis, EoE, lymphocytic esophagitis and healthy controls. C) PCA plot of healthy controls and patients with EoE, non-specific esophagitis and lymphocytic esophagitis, with the exclusion of three outlier patients (N12, N37 and N42). D) Matrix displaying the euclidean distances with unsupervised clustering of rows and columns between patients with non-specific esophagitis, EoE, lymphocytic esophagitis and healthy controls, with the exclusion of three outlier NSE patients (N12, N37 and N42). E) PCA plot of healthy controls and patients with non-specific esophagitis and lymphocytic esophagitis, with the exclusion of three outlier NSE patients (N12, N37 and N42). F) Matrix displaying the euclidean distances with unsupervised clustering of rows and columns between patients with non-specific esophagitis and lymphocytic esophagitis and healthy controls, with the exclusion of three outlier NSE patients (N12, N37 and N42). G) PCA plot of healthy controls and patients with non-specific esophagitis, with the exclusion of three outlier NSE patients (N12, N37 and N42). H) Matrix displaying the euclidean distances with unsupervised clustering of rows and columns between patients with non-specific esophagitis and healthy controls, with the exclusion of three outlier NSE patients (N12, N37 and N42).

**Supplementary figure 4:** Principal component analysis (PC1–PC2 using the top 500 most variable genes) was used to compute Euclidean distances from each Non-Specific Esophagitis (NS) biopsy to the NS centroid (DNS) and the Control centroid (DCTRL). Samples positioned below the diagonal line (DCTRL < DNS) are more transcriptionally similar to Controls (“Control-like”), while samples above the line classify as “NS-like”. The vertical dotted line denotes the 99th percentile of the NS distance distribution, used as a robust cutoff to flag atypical samples. Patient N12, highlighted in red with a black outline, lies beyond this threshold and is therefore identified as an extreme outlier relative to the NS cohort.

**Supplementary figure 5:** PCA plot of healthy control and patients with GERD, non-specific esophagitis and lymphocytic esophagitis, with the exclusion of three outlier NSE patients (N12, N37 and N42).

**Supplementary figure 6:** Bubble plot displaying the complete results of the pathway enrichment analysis performed on the 2152 positively DEGs in non-specific esophagitis compared to healthy controls using the Reactome gene sets. Pathways are ordered by the ratio of DEGs within each gene set to the total number of genes in the set.

|  |  |  |
| --- | --- | --- |
| **Supplementary figure 7:** Heatplot displaying the genes and their expression in Reactome pathways enriched in non-specific esophagitis compared to healthy controls. Pathways are color-coded according to their clustering in the tree plot of Figure 3C. | | |

**3. Supplementary Tables**

Supplementary Table 1: List of 2152 positively differentially expressed genes in patients with non-specific esophagitis vs. healthy controls.

Supplementary Table 2: Hallmark pathway analysis for differentially expressed genes in non-specific esophagitis patients vs. healthy controls.

Supplementary Table 3: Reactome pathways analysis for differentially expressed genes in non-specific esophagitis patients vs. healthy controls.

Supplementary Table 4: List of 51 upregulated genes in non-specific esophagitis patients compared to EoE, lymphocytic esophagitis and healthy controls.

Supplementary Table 5: List of 44 downregulated genes in non-specific esophagitis patients compared to EoE, lymphocytic esophagitis and healthy controls.

**4. References**

1.	Greuter T, Straumann A, Fernandez-Marrero Y, Germic N, Hosseini A, Yousefi S, et al. Characterization of eosinophilic esophagitis variants by clinical, histological, and molecular analyses: A cross-sectional multi-center study. Allergy. 2022;77(8):2520-2533.

2.	Hirano I, Moy N, Heckman MG, Thomas CS, Gonsalves N, Achem SR. Endoscopic assessment of the oesophageal features of eosinophilic oesophagitis: validation of a novel classification and grading system. Gut. 2013;62(4):489-95.

3.	Collins MH, Martin LJ, Alexander ES, Boyd JT, Sheridan R, He H, et al. Newly developed and validated eosinophilic esophagitis histology scoring system and evidence that it outperforms peak eosinophil count for disease diagnosis and monitoring. Dis Esophagus. 2017;30(3):1-8.

4.	<http://www.bioinformatics.babraham.ac.uk/projects/fastqc/>.

5.	Wang L, Wang S, Li W. RSeQC: quality control of RNA-seq experiments. Bioinformatics. 2012;28(16):2184-5.

6.	Kim D, Langmead B, Salzberg SL. HISAT: a fast spliced aligner with low memory requirements. Nat Methods. 2015;12(4):357-60.

7.	Liao Y, Smyth GK, Shi W. featureCounts: an efficient general purpose program for assigning sequence reads to genomic features. Bioinformatics. 2014;30(7):923-30.

8.	Love MI, Huber W, Anders S. Moderated estimation of fold change and dispersion for RNA-seq data with DESeq2. Genome Biol. 2014;15(12):550.
